# Characterization of Novel Bacteriophages for Biocontrol of Bacterial Blight in Leek Caused by *Pseudomonas syringae* pv. *porri*

**DOI:** 10.3389/fmicb.2016.00279

**Published:** 2016-03-15

**Authors:** Sofie Rombouts, Anneleen Volckaert, Sofie Venneman, Bart Declercq, Dieter Vandenheuvel, Camille N. Allonsius, Cinzia Van Malderghem, Ho B. Jang, Yves Briers, Jean P. Noben, Jochen Klumpp, Johan Van Vaerenbergh, Martine Maes, Rob Lavigne

**Affiliations:** ^1^Laboratory of Gene Technology, Department of Biosystems, KU LeuvenLeuven, Belgium; ^2^Unit Plant— Crop Protection, Institute for Agricultural and Fisheries ResearchMerelbeke, Belgium; ^3^Vegetable Research Centre (PCG)Kruishoutem, Belgium; ^4^Research Station for Vegetable ProductionSint-Katelijne-Waver, Belgium; ^5^InagroRumbeke, Belgium; ^6^Laboratory of Applied Microbiology and Biotechnology, Department of Bioscience Engineering, University of AntwerpAntwerpen, Belgium; ^7^Laboratory of Applied Biotechnology, Department of Applied Biosciences, Ghent UniversityGhent, Belgium; ^8^School of Life Sciences, Biomedical Research Institute and Transnational University Limburg, Hasselt UniversityDiepenbeek, Belgium; ^9^Institute of Food, Nutrition and Health, ETH ZurichZurich, Switzerland; ^10^Lab. of Microbiology, Department of Biochemistry and Microbiology, Ghent UniversityGent, Belgium

**Keywords:** *Pseudomonas syringae* pv. *porri*, leek bacterial blight, phage therapy, KIL-like viruses, phylogenomics

## Abstract

*Pseudomonas syringae* pv. *porri*, the causative agent of bacterial blight in leek (*Allium porrum*), is increasingly frequent causing problems in leek cultivation. Because of the current lack of control measures, novel bacteriophages were isolated to control this pathogen using phage therapy. Five novel phages were isolated from infected fields in Flanders (vB_PsyM_KIL1, vB_PsyM_KIL2, vB_PsyM_KIL3, vB_PsyM_KIL4, and vB_PsyM_KIL5), and were complemented with one selected host range mutant phage (vB_PsyM_KIL3b). Genome analysis of the phages revealed genome sizes between 90 and 94 kb and an average GC-content of 44.8%. Phylogenomic networking classified them into a novel clade, named the “KIL-like viruses,” related to the *Felixounalikevirus* genus, together with phage phiPsa374 from *P. syringae* pv. *actinidiae*. *In vitro* characterization demonstrated the stability and lytic potential of these phages. Host range analysis confirmed heterogeneity within *P. syringae* pv. *porri*, leading to the development of a phage cocktail with a range that covers the entire set of 41 strains tested. Specific bio-assays demonstrated the *in planta* efficacy of phages vB_PsyM_KIL1, vB_PsyM_KIL2, vB_PsyM_KIL3, and vB_PsyM_KIL3b. In addition, two parallel field trial experiments on three locations using a phage cocktail of the six phages showed variable results. In one trial, symptom development was attenuated. These data suggest some potential for phage therapy in controlling bacterial blight of leek, pending optimization of formulation and application methods.

## Introduction

In recent years, an increase in the prevalence of leek bacterial blight was noted in Flanders, Belgium. The disease is caused by the bacterial pathogen *Pseudomonas syringae* pv. *porri*, which was first reported in the United Kingdom by Lelliott (1952). Two decades later, the species was isolated in New Zealand by Hale (1975) who further described the pathogen. Later, the bacteria was classified as a new pathovar, *P. syringae* pv. *porri*, based on extensive research by Samson et al. in France in 1998. Currently, the disease has spread globally and is reported in The Netherlands (Janse, 1982; van Overbeek et al., 2010), Italy (Varvaro, 1983), the United States (Koike et al., 1999), Australia (Noble et al., 2006), Greece (Glynos and Alivizatos, 2006), Japan (Goto, 1972), and Korea (Myung et al., 2011, 2012). Typical symptoms include leaf curling and yellowing of the middle vein in young plants and water soaked spots on older leaves and flowering stems (Samson et al., 1998; Noble et al., 2006). Leek (*Allium porrum*) is the major host, but the pathogen has also been diagnosed on onion (*Allium cepa*) and shallot (*Allium cepa* var. *aggregatum*; Noble et al., 2006). The disease is known to be transmitted by seed (Koike et al., 1999; Ikene et al., 2003), but crop waste also plays a role in contaminating new leek plants in the field (van Overbeek et al., 2010).

The recent rise in leek bacterial blight is consistent with a growing number of Flemish farmers obtaining leek transplants from plant nurseries. High plant densities in those nurseries, combined with plant manipulations such as irrigation and mowing, promote dissemination of the pathogen among the transplants (Koike et al., 1999). In a previous study, the causative agent of recent leek blight epidemics in Flanders was examined, leading to the proposed subdivision of the strains into two groups, with small genomic differences (Rombouts et al., 2015). Knowledge on this intra-pathovar diversity is important to determine the specificity of phages isolated and characterized within this study.

To date, disease management in leek production mainly consists of prevention. Clean seeds, a strict hygiene in plant nurseries and the use of more tolerant varieties are important control measures. However, once the first symptoms of bacterial blight appear, no solution is available because of the ban on streptomycin derivatives to control bacterial plant diseases in the European Union. The use of copper-based agrichemicals is also undesirable in view of ecotoxicity and bacterial resistance development (Cooksey, 1994; Pietrzak and McPhail, 2004; Hwang et al., 2005). Therefore, the use of phage therapy constitutes an attractive alternative. Bacteriophages are viruses that specifically infect bacteria, their replication resulting in the lysis of their bacterial host and the release of newly formed viral particles. Several reviews have previously been published, highlighting the possibilities and limitations of phage therapy in plant disease control (Gill and Abedon, 2003; Jones et al., 2007; Balogh et al., 2010). In agriculture, phage therapy research has been conducted for pathogens in other crops including “*Dickeya solani*” (Adriaenssens et al., 2012), *Erwinia amylovora* (Gill et al., 2003; Boulé et al., 2011), *Pectobacterium carotovorum* (Ravensdale et al., 2007), *Ralstonia solanacearum* (Fujiwara et al., 2011), *Xanthomonas axonopodis* pvs. *citri* and *citrumelo* (Balogh et al., 2008), and *P. syringae* pv. *actinidiae* (Frampton et al., 2014). To date, phage research resulted in a limited number of commercial phage-based products for agricultural use. These products are for control of two tomato pathogens, *Xanthomonas campestris* pv. *vesicatoria* and *P. syringae* pv. *tomato* (AgriPhage; Flaherty et al., 2000; Balogh et al., 2003) and against potato rot (Biolyse, APS Biocontrol Ltd.).

Phages infecting *P. syringae* pv. *porri* have not yet been described. In this manuscript, we focus on the isolation of novel phages which cover the bacterial diversity present in Flemish leek production. Extensive *in vitro* characterization of the phages was followed by bio-assays and field trials to evaluate the potential of these phages for the biocontrol of bacterial blight in leek.

## Materials and methods

### Bacterial strains and growth conditions

The bacterial strains used in this study are listed in Table 1. *P. syringae* pv. *porri* strains were isolated from fields in Flanders and covered the existing diversity. Their identification and characterization was previously published (Rombouts et al., 2015). These isolates were supplemented with P55, an isolate from soil from the study of van Overbeek et al. (2010), and reference strains of *P. syringae* pv. *porri* from the Collection Française de Bactéries Phytopathogènes (CFBP; Beaucouzé, France), including the type strain and a set of phylogenetic related strains. Bacteria were grown in lysogeny broth with reduced salt concentration (LB_rs_) (0.5 g/l NaCl) at 26°C, or on LB_rs_ plates supplemented with 1.5% agar. LB_rs_ with 0.7% agar (soft agar) was used for the plate overlays.

**Table 1:** *****P. syringae*** pv. ***porri*** strains and related ***P. syringae*** pathovars with their phage sensitivity and BOX-PCR profiles**.

**Bacterial strain^a^**	**Plant origin**	**Origin^b^**	**Year^c^**	**Identification**	**KIL 1**	**KIL 2**	**KIL 3**	**KIL 4**	**KIL 5**	**KIL 3b**	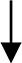	**BOX-PCR**
•GBBC 1427	*Allium porrum*	BE	2012	*P.s*. pv. *porri*	−	−	−	+	+	−	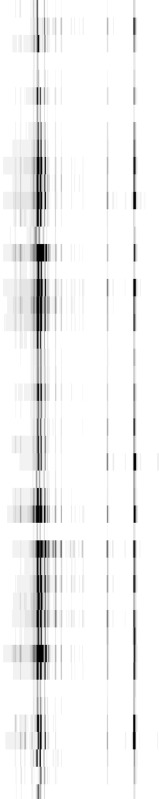	
•GBBC 1428	*Allium porrum*	BE	2012	*P.s*. pv. *porri*	−	−	−	+	+	−		
•GBBC 1438	*Allium porrum*	BE	2012	*P.s*. pv. *porri*	−	−	−	+	+	−		
•GBBC 1444	*Allium porrum*	BE	2012	*P.s*. pv. *porri*	−	−	−	+	+	−		
•LMG 28496	*Allium porrum*	BE	2012	*P.s*. pv. *porri*	−	−	−	+	+	−		
×GBBC 715	*Allium porrum*	BE	2001	*P.s*. pv. *porri*	+	+	+	+	+	+		
×GBBC 722	*Allium porrum*	BE	2001	*P.s*. pv. *porri*	+	+	+	+	+	+		
×GBBC 728	*Allium porrum*	BE	2002	*P.s*. pv. *porri*	+	+	+	+	+	+		
×GBBC 747	*Allium porrum*	BE	2002	*P.s*. pv. *porri*	+	+	+	+	+	+		
×GBBC 1088	*Allium porrum*	MA	2011	*P.s*. pv. *porri*	+	+	+	+	+	+		
×GBBC 1089	*Allium porrum*	MA	2011	*P.s*. pv. *porri*	+	+	+	+	+	+		
×GBBC 1090	*Allium porrum*	MA	2011	*P.s*. pv. *porri*	−	−	−	−	−	+		
×GBBC 1113	*Allium porrum*	BE	2003	*P.s*. pv. *porri*	+	+	+	+	+	+		
×GBBC 1165	*Allium porrum*	BE	2004	*P.s*. pv. *porri*	+	+	+	+	+	+		
×GBBC 1166	*Allium porrum*	BE	2004	*P.s*. pv. *porri*	+	+	+	+	+	+		
×GBBC 1170	*Allium porrum*	BE	2004	*P.s*. pv. *porri*	+	+	+	+	+	+		
×GBBC 1184	*Allium porrum*	BE	2004	*P.s*. pv. *porri*	+	+	+	+	+	+		
×GBBC 1255	*Allium porrum*	BE	2005	*P.s*. pv. *porri*	+	+	+	+	+	+		
×GBBC 1256	*Allium porrum*	BE	2005	*P.s*. pv. *porri*	−	−	−	−	−	+		
×GBBC 1267	*Allium porrum*	BE	2005	*P.s*. pv. *porri*	+	+	+	+	+	+		
×GBBC 1269	*Allium porrum*	BE	2005	*P.s*. pv. *porri*	−	−	−	−	+	+		
×GBBC 1272	*Allium porrum*	BE	2005	*P.s*. pv. *porri*	+	+	+	+	+	+		
×GBBC 1273	*Allium porrum*	BE	2005	*P.s*. pv. *porri*	+	+	+	+	+	+		
×GBBC 1277	*Allium porrum*	BE	2006	*P.s*. pv. *porri*	+	−	−	−	−	+		
×GBBC 1286	*Allium porrum*	BE	2006	*P.s*. pv. *porri*	+	+	+	+	+	+		
×GBBC 1311	*Allium porrum*	BE	2007	*P.s*. pv. *porri*	+	+	+	+	+	+		
×GBBC 1424	*Allium porrum*	BE	2012	*P.s*. pv. *porri*	+	+	+	+	+	+		
×GBBC 1426	*Allium porrum*	BE	2012	*P.s*. pv. *porri*	+	+	+	+	+	+		
×GBBC 1433	*Allium porrum*	BE	2012	*P.s*. pv. *porri*	+	+	+	+	+	+		
×GBBC 1434	*Allium porrum*	BE	2012	*P.s*. pv. *porri*	+	+	+	+	+	+		
×GBBC 1435	*Allium porrum*	BE	2012	*P.s*. pv. *porri*	+	+	+	+	+	+		
×GBBC 1452	*Allium porrum*	BE	2012	*P.s*. pv. *porri*	+	+	+	+	+	+		
×GBBC 1459	*Allium porrum*	BE	2013	*P.s*. pv. *porri*	+	+	+	+	+	+		
×GBBC 1462	*Allium porrum*	BE	2013	*P.s*. pv. *porri*	+	+	+	+	+	+		
×GBBC 1893	*Allium porrum*	NL	2013	*P.s*. pv. *porri*	+	+	+	+	+	+		
×GBBC 1894	*Allium porrum*	NL	2013	*P.s*. pv. *porri*	+	+	+	+	+	+		
×LMG 28495	*Allium porrum*	BE	2011	*P.s*. pv. *porri*	+	+	+	+	+	+		
×P55^d^	soil of leek field	NL	2010	*P.s*. pv. *porri*	+	−	−	−	−	+		
×CFBP 1908^PT^	*Allium porrum*	FR	1978	*P.s*. pv. *porri*	+	+	+	+	+	+		
×CFBP 1687	*Allium porrum*	GB	1949	*P.s*. pv. *porri*	+	+	+	+	+	+		
×CFBP 1770	*Allium porrum*	NZ	1973	*P.s*. pv. *porri*	+	+	+	+	+	+		
CFBP 3228^PT^	*Oryza sativa*	JP	1983	*P.s*. pv. *oryzae*	−	−	−	−	−	−		
CFBP 1634^PT^	*Coffea arabica*	BR	1958	*P.s*. pv. *garcae*	−	−	+	−	−	+		
CFBP 1674^PT^	*Avena sativa*	−	1958	*P.s*. pv. *striafaciens*	−	−	−	−	−	−		
CFBP 2216^T^	*Avena sativa*	GB	1958	*P.s*. pv. *coronafaciens*	−	−	−	−	−	−		
CFBP 4117^PT^	*Zizania aquatica*	US	1983	*P.s*. pv. *zizaniae*	−	−	−	−	−	−		

*The two symbols (• and ×) indicate two BOX-PCR pattern groups. The arrow gives the position of the discriminative band. Light and dark green: lysis after 24 and 48 h, respectively and yellow: lysis from without*.

a *GBBC: culture collection of plant pathogenic bacteria at ILVO; CFBP, Collection Française de Bactéries Phytopathogènes; INRA Angers; LMG, Belgian Coordinated Collections of Microorganisms at the Laboratory of Microbiology of Ghent University with ^T^ as type strains and ^PT^ as pathovar reference strains*.

b *Geographical origin*.

c *Year of isolation*.

d *received from van Overbeek et al. (2010)*.

### Bacteriophage isolation, amplification, and purification

Phages were isolated from soil samples taken in 2011 and 2012, from the same fields from which the *P. syringae* pv. *porri* strains were isolated. To enrich for phages, a bacterial host (LMG 28495 or LMG 28596) was grown in 25 ml LB_rs_ at 26°C up to exponential growth phase and 5 g of soil sample was added. After overnight incubation, 250 μl chloroform was added and incubation was continued for 1 h to lyse all bacterial cells. Next, the mixture was centrifuged (30 min, 4500 rpm) in a Sorvall Legend RT+ centrifuge with swing-out 4-place rotor, type 75006445 (Thermo Scientific, Waltham, MA, USA). The supernatant was filtered through a Millex® syringe filter with a 0.45 μm pore size (Merck Millipore Ltd.) and 10 μl was spotted on a soft agar layer that contained the bacterial host. Lysis zones were picked up with sterile toothpicks and suspended in phage buffer (10 mM Tris-HCl; pH 7.5; 10 mM MgSO_4_; 150 mM NaCl). These suspensions were plated by pooling 250 μl overnight bacterial host culture, 100 μl phage suspension and 4 ml LB_rs_ overlay agar, according to the overlay agar technique (Adams, 1959). After overnight incubation at 26°C, single plaques were picked up again. Three successive single plaque isolations were performed to achieve pure phage isolates. Based on their DNA restriction pattern (data not shown) different phages were selected for further analysis.

One bacteriophage (KIL3b) was obtained after a co-cultivation experiment. This experiment consisted of the infection of an exponentially growing liquid culture of *P. syringae* pv. *porri* strain CFBP 1687 with phage KIL3 at a multiplicity of infection (MOI) of 0.01. For 4 weeks, 40 μl of the liquid culture was transferred to a tube with 4 ml fresh LB_rs_ medium every 2 to 3 days and incubated at room temperature. Supernatant of every two-day-old liquid culture was spot tested against the bacterial strain P55 to select for a broadened lytic activity as none of the other phages were able to lyse this strain.

Phages were amplified by plating 10^5^ plaque forming units (pfu) per plate together with their bacterial host on a soft agar overlay plate. After overnight incubation at 26°C, the soft agar was scraped off and suspended in phage buffer. Soft agar and cell debris were subsequently removed by centrifugation (20 min, 4000 rpm) in a Sorvall Legend RT+ centrifuge. The supernatant was filtered over a 0.22 μm membrane Millex® syringe filter (Merck Millipore Ltd.), after which polyethylene glycol (PEG) 8000 (Sigma-Aldrich) containing 1 M NaCl was added to the suspension to a final concentration of 8%_w∕v_. After minimum 3 h incubation at 4°C, phages were precipitated by centrifugation (30 min, 4600 rpm) and the pellet was dissolved in 1 ml phage buffer.

Phage purification was performed using CsCl-gradient ultracentrifugation. A phage suspension (15 ml) with a minimum concentration of 10^10^ pfu/ml was layered on a CsCl step gradient (1.33, 1.45, 1.50, and 1.70 CsCl g/cm^3^) and centrifuged (3 h, 140.000 × g) with a Beckman Coulter Optima L-90K ultracentrifuge (rotor type SW28) (Brea, CA, USA). All phages were collected at the interface between the 1.45 and 1.50 g/cm^3^ density layer and dialyzed three times for 30 min against 300 volumes of phage buffer.

### Electron microscopy

To obtain transmission electron microscopy (TEM) images of the bacteriophages, dilutions of the samples were spotted on carbon coated grids (Quantifoil, Großlöbichau, Germany) after glow-discharge, and negatively stained with 2% uranyl acetate. A Philips CM12 microscope was used at 120 kV acceleration voltage. Images were produced using a Gatan Orius 1 k camera.

### Host range analysis and general characterization

Phage host range was tested by spotting a phage suspension on plates with a soft agar layer supplied with a specific bacterial isolate. For all the phages, three different concentrations, ranging from 10^2^ to 10^6^ pfu/ml, were tested to determine infectivity. The bacterial strains used in the host range assay are listed in Table 1.

Adsorption experiments were performed according to Adriaenssens et al. (2012). Briefly, bacteria and phage were mixed at an MOI of 0.0001. Next, samples were taken every minute and bacteria were immediately lysed by adding chloroform. The upper phase was titred to determine the number of non-adsorbed phages.

Killing curves at different MOIs were generated by infecting a bacterial culture at an optical density at 600 nm (OD_600_) of 0.3. The OD_600_ of infected and uninfected cultures was monitored every 20 min during 6 h. OD_600_ results were the average of three independent biological repeats.

Phage stability was tested by incubating a phage suspension of 10^6^ pfu/ml in phage buffer at different temperatures (−20, 4, 16, 30, 37, and 50°C) and at different pH ranging from 1 to 13 (universal buffer consisting of 150 mM KCl, Janssen Chimica, Geel, Belgium; 10 mM KH_2_PO_4_, VWR International, Leuven, Belgium; 10 mM sodium citrate, Acros Organics; 10 mM H_3_BO_3_, Acros Organics; adjusted to pH 1–13 with NaOH, or HCl). Infectivity of the phages at different temperatures was tested by incubating soft agar plates with spots of different phage concentrations at room temperature (±21°C), 26 and 30°C and counting the infective phage titers.

The frequency of bacterial resistance was determined using an adaptation of a previously developed method (Beale, 1948). Using the agar overlay method, phage and bacteria were plated at MOI 1 and MOI 10, in order to lyse all bacteria on the plate. After 72 h of incubation, emerging colonies were counted and about 20 colonies were cultured and re-tested for resistance against the phages in Table 2. Phage-resistant isolates were plated on Pseudomonas agar F (PAF, Becton Dickinson) supplemented with glycerol to check for fluorescence under UV-light and analyzed with a multiplex PCR containing specific primers (Ineke Wijkamp, Rijkzwaan, personal communication) to confirm their *P. syringae* pv. *porri* identity.

**Table 2:** **Bacterial host of the new bacteriophages and phage genome characteristics determined by bioinformatic analysis**.

**Phage name**	**GenBank accession number**	**Bacterial host**	**Genome length (bp)**	**GC content (%)**	**# ORFs**	**# tRNAs**	**Terminators**	**Bacterial promoters**
KIL1	KU130126	CFBP 1687	90695	44.86	159	5	18	57
KIL2	KU130127	CFBP 1687	92466	44.79	163	9	17	59
KIL3	KU130128	CFBP 1687	92089	44.74	161	5	17	61
KIL4	KU130129	LMG 28496	92825	44.89	167	9	18	60
KIL5	KU130130	LMG 28496	93384	44.97	169	9	18	59
KIL3b	KU130131	CFBP 1687	92099	44.72	161	5	17	61

Lysogeny in these resistant bacteria was tested as previously described (Petty et al., 2006). Supernatant from overnight cultures of resistant bacteria were collected and tested for the spontaneous release of phage particles by spotting 5 μl drops onto soft agar containing the different bacterial hosts. The supernatants from CFBP 1687 and LMG 28496 were used as negative controls, and pure phage solutions (10^6^ pfu/ml) were used as positive controls for plaque formation.

### Genome and proteome analysis

#### DNA isolation and sequencing

Contaminating bacterial DNA was removed from phage lysates by DNase treatment. Upon inactivation of the DNase by the addition of EDTA, phage particles were disrupted by adding SDS and proteinase K. The released phage DNA was purified by standard phenol-chloroform extraction and subsequent ethanol precipitation according to Sambrook and Russell (2001). DNA concentration and purity was verified with the NanoDrop ND-1000 spectrophotometer (Thermo Fisher Scientific Inc.). DNA integrity and genome size was evaluated on an 0.8% agarose gel. High throughput sequencing was done using the Illumina MiSeq platform. A 2^*^250 bp paired-end library was prepared for each sample, tagged with a unique adapter sequence. The quality of each library preparation was controlled using the Agilent Bioanalyzer. All library preps were equally pooled and sequenced. After processing, the reads of every library prep were assembled in a single contig with a general coverage over 1000x using the CLC Bio Genomics Workbench de novo assembly algorithm (version 7.5.1) (Aarhus, Denmark).

#### “*In silico*” analysis

The genomes were scanned for open reading frames (ORFs) with GeneMark.hmm and GeneMarkS software (Lukashin and Borodovsky, 1998; Besemer et al., 2001). Shine-Dalgarno sequences were verified manually upstream of each annotated ORF. Functional annotation was carried out by comparing translated ORFs in a Blastp analysis (Altschul et al., 2005) against the nonredundant GenBank protein database and with the Protein Homology/analogY Recognition Engine v2.0 (PHYRE2) (Kelley and Sternberg, 2009). tRNAs were detected with the programs ARAGORN and tRNAscanSE (Lowe and Eddy, 1997; Laslett and Canback, 2004). Rho-independent terminators were predicted with ARNold, a search program that combines two complementary programs: RNAMotif and Erpin (Gautheret and Lambert, 2001; Macke et al., 2001). Probable promoter sequences were identified by looking for conserved intergenic motifs in the 100 bp upstream of every ORF with MEME and PHIRE (Lavigne et al., 2004; Bailey et al., 2009).

#### Protein clustering and phylogenetic analysis

Homologous proteins, deduced from their DNA sequences, were examined in the phages KIL1, KIL2, KIL3, KIL4, KIL5, KIL3b, and a number of fully sequenced bacteriophages using HMMER (version3.0; http://hmmer.janelia.org), together with the ACLAME search (version0.4; http://aclame.ulb.ac.be; as of June 2015). In this manner, we retrieved a total of 4632 predicted protein sequences represented in the genomes of the 26 relevant phages (Table S2). We performed pairwise similarity comparisons for each predicted protein by using the ACLAME database with the database of “viruses” and an *E*-value < 0.001 (Lima-Mendez et al., 2008). The remaining proteins which could not be assigned into any ACLAME protein family were defined as the unclassified protein families (UPFs) as previously described (Jang et al., 2013). The protein families that were common to all 32 representative phage genomes were identified and their phylogenetic relationships were inferred. After multiple sequence alignment of the conserved proteins using CLUSTALW2, non-informative positions were excluded with the BMGE software package (Criscuolo and Gribaldo, 2010). The two alignments were then concatenated into a FASTA file and a phylogenetic tree was built with MEGA5 (version 5.2.1; http://www.megasoftware.net/; Tamura et al., 2011) using a Jones–Taylor–Thornton (JTT) model. To estimate the robustness of the trees, we used the maximum-likelihood algorithm provided with bootstrap support (*n* = 1000 replicates).

#### Proteome analysis

Structural proteins of phages KIL3 and KIL5 were identified by SDS-PAGE gel electrophoresis, isolation of gel bands, and subsequent trypsinization and ESI-MS/MS as described previously (Lavigne et al., 2009a). The resulting data were analyzed using Mascot (version 2.3.01) and Sequest (version 1.2.0.208), against a database containing all possible ORFs based on the DNA sequence of the phages.

### Phage therapy experiments

#### Bio-assay

*In planta* activity of the phages was tested by injecting phage and bacterial suspensions into leek leaves. Leek plants of the disease susceptible cultivar Krypton (Nunhems) were grown in separate pots until full development of three leaves. Next, 0.1 ml of bacterial suspension was injected with a syringe into the middle of the leaf. Subsequently, 3 cm above the bacterial injection place 0.1 ml of phage suspension (10^9^ pfu/ml) was injected (Supplementary Figure 1). In the first bio-assay all 41 *P. syringae* pv. *porri* strains (10^7^ CFU/ml) were inoculated in leek leaves (three inoculated leaves/strain) and all phage types (10^9^ pfu/ml) were co-inoculated with a *P. syringae* pv. *porri* representative (eight inoculations/phage-bacteria combination). This experiment was repeated using the disease susceptible cultivar Striker (Bejo) and a lower bacterial concentration (10^6^ CFU/ml). Phages KIL1, KIL2, KIL3, and KIL3b (10^9^ pfu/ml) were co-inoculated with bacterial strain CFBP 1687 and phages KIL4 and KIL5 with LMG 28496 (10 inoculations/phage-bacteria combination). Plants were covered for 48 h with a plastic bag to maintain humid conditions and kept at a temperature of 25°C. Lesion lengths were measured after 10 days. Injections with only bacteria, phage or buffer served as controls.

#### Field trials

In a first trial, leek transplants were treated with phages before they were planted in an infested field. First, the field was infested by spraying a solution of CFBP 1687 with a concentration of 10^6^ CFU/ml on the soil at a rate of 1000 l/ha, at 1.5 bar and with 0.01% Tween® 20 as surfactant. The next day, before planting, leek plants were submerged in a solution containing a mixture of the six different phages, each at a concentration of 10^7^ pfu/ml. Control plants were planted without treatment. Four blocks of the phage-treated and non-treated plants were randomly distributed over the field, with each block containing 300 plants. At multiple time points after planting, disease incidence (number of damaged plants) as well as disease severity (% of leaf surface affected) was measured for 20–50 plants per block. This trial was performed simultaneously on three different locations in Flanders: Kruishoutem, PCG (N 50.94337°, E 3.52710°), Sint-Katelijne Waver, PSKW (N 51.078120°, E 4.528180°), and Beitem, Inagro (N 50.901508°, E 3.124464°).

In a second trial, leek transplants were planted in a non-infested field. Two months after planting, bacterial infection was performed with strain CFBP 1687. A suspension with a concentration of 10^6^ CFU/ml was spread over the plants at a rate of 1000 l/ha, at 1.5 bar and with 0.01% Tween® 20 as surfactant. The next day, a mix of the six different phages, each at a concentration of 10^9^ pfu/ml and supplemented with 0.01% Tween® 20, was sprayed over the plants, in a way that each plant was covered with on average 10^8^ pfu of each phage after spraying. Plants were covered with plastic for 48 h and assessed as described. This trial was also performed in parallel on the three different locations in Flanders mentioned above.

#### Statistical analysis of data

Statistical analyses were performed with IBM SPSS Statistics 20. For the bio-assays, normality of the data was assessed with the Kolmogorov-Smirnov and Shapiro-Wilk test at a significance level of 0.05. Homogeneity of variances was tested using the Levene's test. When the data were distributed normally and homoscedasticity was proven, an one-way ANOVA test was performed to check for differences. If the tests showed significant differences, the data were further analyzed with a *post-hoc* Tukey HSD test. To test for differences between groups of not normally-distributed data, a Kruskal-Wallis non-parametric test was used. When differences were present, Mann-Whitney U non-parametric test was used to compare the groups, paired two by two. For the field trials, the statistical program ARM (version 2015.3) was used. This program, which is designed specifically for field trials, uses the Bartlett's test to test the homogeneity of variances and the Kurtosis and Skewness tests to check normality. One-way ANOVA with the *post-hoc* Duncan test was used for normal distributed and homoscedastic data. As a non-parametric test, the Friedman test was applied to check for differences between several variables.

## Results

### Phage isolation and TEM

Phages were isolated from soil samples taken in 2011 and 2012 from the same fields from which *P. syringae* pv. *porri* isolates were obtained (Table 2). After enrichment of the phages present in the soil, their capacity to lyse *P. syringae* pv. *porri* strain CFBP 1687 was tested. Based on their differential DNA restriction pattern (data not shown) phages were selected for further analysis. By this method, five novel phages were discovered for *P. syringae* pv. *porri* and the phages were named KIL1, KIL2, KIL3, KIL4, and KIL5 (referring to the involved research institutes KU Leuven and ILVO) with scientific names vB_PsyM_KIL1 etc. as proposed by Kropinski et al. (2009). The host strains used for amplification and characterization of each of these phages are indicated in Table 2.

**Figure 1:**
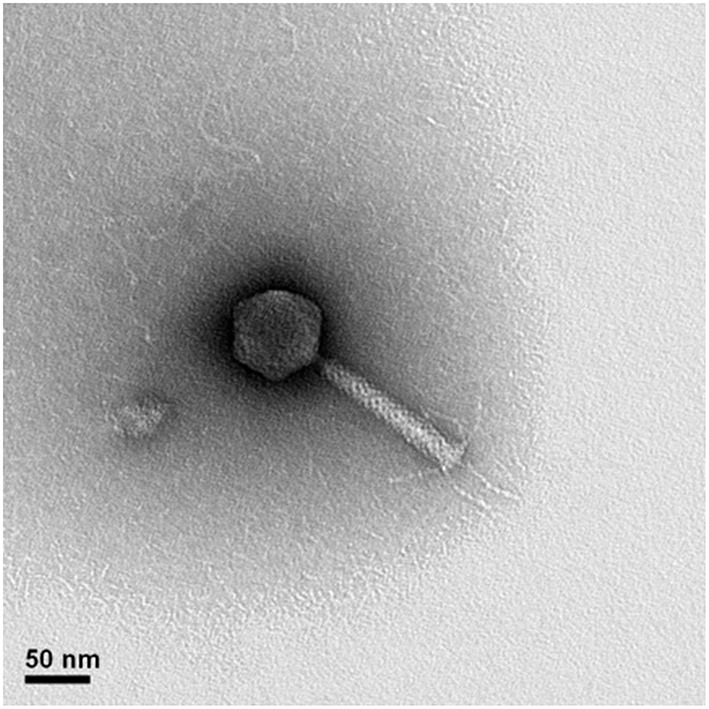
**EM picture of phage KIL1**.

TEM images of a phage representative revealed that they belong to the *Myoviridae* family with morphological similarity to the Pseudomonas phage *PB1* (genus *Pbunalikevirus*), as shown for phage KIL1 in Figure 1. The other analyzed phages have an identical morphology.

### Host range analysis

To investigate the specificity of the phages, a host range analysis was performed (Table 1), by using standard serial dilution spotting assays to distinguish infection from lysis from without phenomena, as described previously (Adriaenssens et al., 2012). Some of the bacterial isolates could not be infected by our five phage isolates from soil, therefore the host-range mutant phage KIL3b was developed. The results indicate that the bacterial strains can be divided in three groups. One group consists of five field isolates from the year 2012 and was only infected by phages KIL4 and KIL5. A second group of strains was infected by all the investigated phages and a third group of strains only showed lysis after 48 h and by a limited number of phages. None of the pathovars related to *P. syringae* pv. *porri* was infected by the phages demonstrating their specificity for pathovar *porri*. However, *P. syringae* pv. *garcae* (CFBP 1634) showed “lysis from without” at phage concentrations of 10^6^ pfu/ml. In addition, none of the phages infected all *P. syringae* pv. *porri* strains tested but the combined host range of the phages covers all 41 isolates tested. A previous BOX-PCR analysis on DNA of all the bacterial strains used in the host range analysis provided information about their genomic diversity and identified two groups within *P. syringae* pv. *porri* (Rombouts et al., 2015). These two groups were confirmed by phage profiling, suggesting that differences in the bacterial genome are responsible for the observed host range. Two phages, KIL3 and KIL5 were selected for further characterization.

### General characteristics

To assess the infection parameters, adsorption experiments were performed for phage KIL3. On average, 84% of phages were irreversibly adsorbed to the host cell after 1 min and 99% after 6 min with an adsorption constant k [k = (2.3/(B^*^t))^*^log(P_0_/P), with B the bacterial titer at time zero, P the phage titer and t the time] at 1 min of 7.52 × 10^−9^ ml/min. Compared to other *Myoviridae* phages, this is slower than reported for phage T4 (2.4 × 10^−9^ ml/min) but comparable to the adsorption constants of phages LIMEstone1 (9.53 × 10^−9^ ml/min) and LIMEstone2 (2.05 × 10^−8^ ml/min) infecting *Dickeya* (Kasman et al., 2002; Adriaenssens et al., 2012).

Killing curves generated by infecting an exponentially growing cultures of *P. syringae* pv. *porri* CFBP 1687 and LMG 28496 with phages KIL3 and KIL5, respectively, at different MOI demonstrated their virulence (Supplementary Figure 2). Optical density of the bacterial cultures at 600 nm decreased after 80 and 100 min for phage KIL3 and KIL5, respectively. KIL5 showed a later but steeper decline reaching OD_600_ < 0.1 after 180 min instead of 220 min for KIL3. No rise in OD_600_ appeared within the 5 h of monitoring indicating that resistance did not develop at that point.

The influence of temperature and pH on the viability was tested. As shown for KIL3 and KIL5 (representatives of the two phage clades in the cocktail), the phages were stable between 4 and 37°C in phage buffer for 24 h, but after 24 h of incubation at 50°C, a two and one log_10_ unit decrease was noted for phage KIL3 and KIL5, respectively. After freezing, all viable KIL3 phages were lost, while the titer of KIL5 decreased by three log10 units. Phages were stable from pH 4 to 12 for 24 h.

An analysis of the optimal infection temperature revealed that none of the phages are able to infect their host when grown at temperatures of 30°C. Phages KIL1, KIL2, KIL3, KIL4, and KIL5 were able to infect at temperatures of 26°C and room temperature (±21°C), and KIL3b only infected at room temperature.

For two phage-host interactions, frequency of bacterial resistance development was determined. An exponentially growing culture of strain CFBP 1687 was infected with KIL3 at MOI 1 and after 72 h incubation, an average of 110 phage-resistant bacteria appeared on plate. To exclude the possibility of contamination, the identity of 12 colonies was verified with PCR-analysis using specific primers, confirming their identity as *P. syringae* pv. *porri*. This resulted in a resistance frequency of 1.83 × 10^−6^. A host screen with those resistant bacterial derivatives revealed that five of them were resistant to the six phages, a phenomenon called cross-resistance. The other isolates could still be infected by the host-range mutant phage KIL3b. The same experiment was performed with phage KIL4. After 72 h incubation on average 200 resistant colonies appeared and PCR-analysis was used to confirm the identity of 12 of them, resulting in a calculated resistance frequency of 3.33 × 10^−6^. Again, cross-resistance was observed for part of the strains (four out of 12), the other resistant strains were still infected by KIL3b.

A spot test with supernatant from an overnight culture of those resistant isolates on strains CFBP 1687 and LMG 28496 suggested there was no induction of lysogens under the conditions tested. In general, as all phages produced clear plaques there was no indication for lysogeny.

### Genome and proteome analysis

#### Genome analysis

The genomes of all six phages were sequenced using the Illumina MiSeq platform and single contigs were obtained. Information about the genome characteristics of the phages is summarized in Table 2.

**Figure 2:**
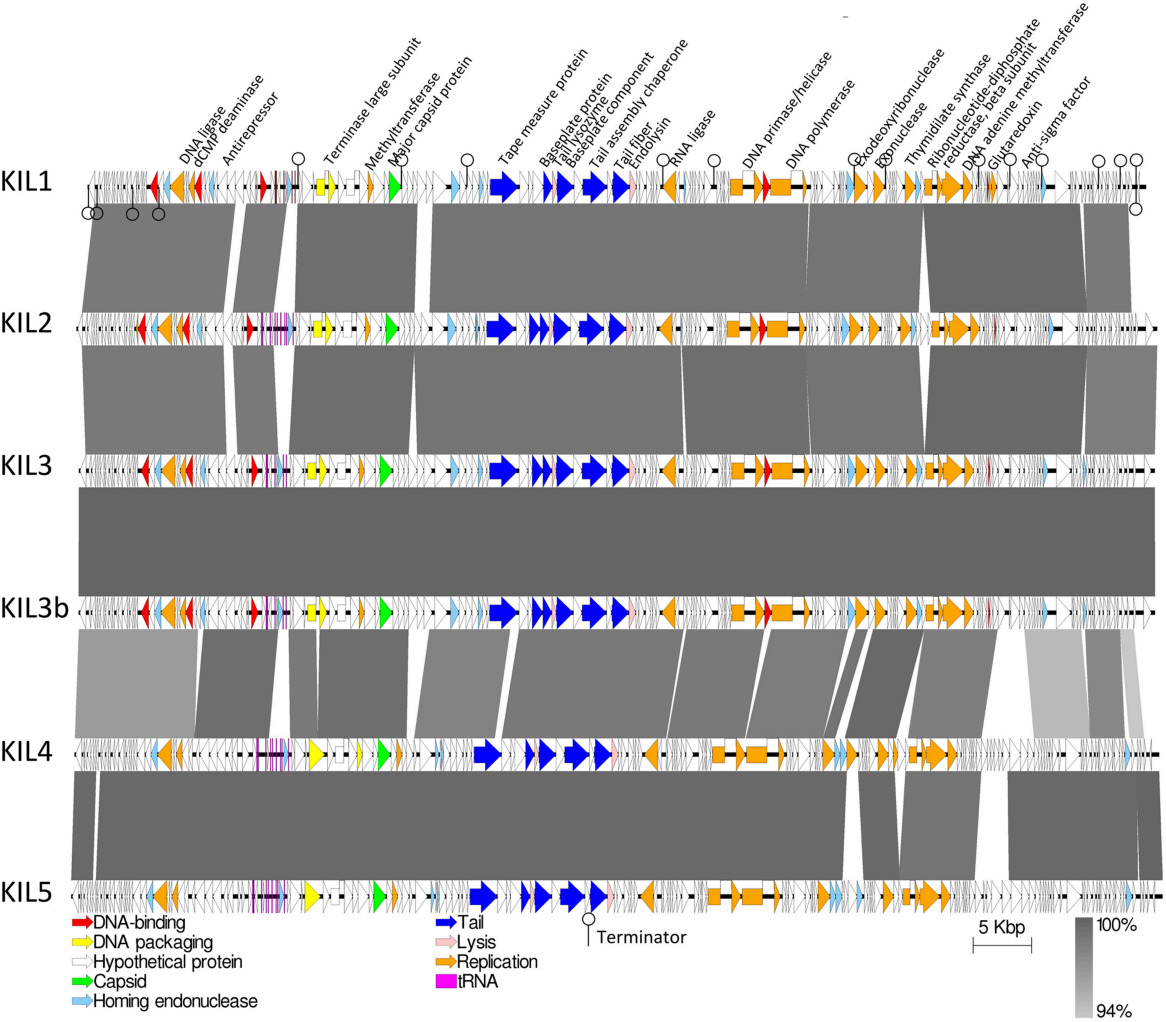
**Representation of the genome organization of the six KIL-like phages and homology (blastn) between them**.

ORFs were identified and similarity at the protein level was verified by Blastp analysis. In addition, a putative function could be assigned to some ORFs without homologs using PHYRE2. All tRNAs were found in the same region spanning 2.7 kb in front of the large terminase subunit, a part of the packaging machinery (Figure 2). This is consistent with the genome organization of the related PAK_P1-like phages (Debarbieux et al., 2010; Henry et al., 2015). When comparing the tRNAs present in the six genomes, phages KIL1, KIL3, and KIL3b encode the same five tRNAs. KIL2, KIL4, and KIL5 also have these five tRNAs in their genomes, supplemented with four additional tRNAs. Depending on the phage, 17 or 18 rho-independent terminators were retained after manual verification and were located across the entire genome. Probable promoter sequences were identified by looking for conserved motifs in the 100 bp upstream region of every ORF. Using MEME, a motif resembling a typical *E. coli* bacterial promoter sequence (TTGACA-N_17−18_-TATAAT) was found in all phages. As in the most closely related *P. syringae* phage PhiPsa374, the 41 bp conserved motif surrounding the bacterial promotor sequences in PAK_P1-like viruses was not encountered thereby confirming its specificity for *P. aeruginosa* phages (Henry et al., 2015). No toxin genes, virulence genes or genes related to lysogeny were discovered in the phage genomes, indicating their suitability for phage therapy.

A remarkable feature of all KIL-like phages is the presence of a homopolymeric G-stretch of variable length at one or two locations in the genome (Table S1). Illumina reads at those positions contain 6–13 Gs. Both stretches are found in intergenic regions, yet not clearly associated to known promoter elements. A similar feature was reported previously in PB1-like phages, in a sequence coding for a baseplate protein and in the ORF coding for a DNA adenine methylase in Bordetella phages BPP-1, BIP-1, and BMP-1 (Liu et al., 2004; Ceyssens et al., 2009). It was proposed that the resulting frameshift serves as a control point for expression levels of the protein. Another remarkable region in the KIL-like phage genomes is a long A-stretch varying from 14 to 33 nucleic acids in length. In the most related phage phiPsa374 those homopolymeric stretches are not present. A phylogenetic analysis of the six phages based on their genome sequences confirms the subdivision of the phages into two groups, correlated to their host range. The first group constitutes of phage KIL1, KIL2, KIL3, and KIL3b, the second group contains phages KIL4 and KIL5. Between the groups, some differences in the ORFs are predicted, mostly among unknown genes. The difference between the genomes of phage KIL3 and KIL3b, which is a host range mutant of the former, is limited to the number of repeats located in the homopolymeric A- and G-stretches. No clear biological function could be attributed to this phenomenon.

#### The phylogenetic and proteome-based relationships

**Figure 3:**
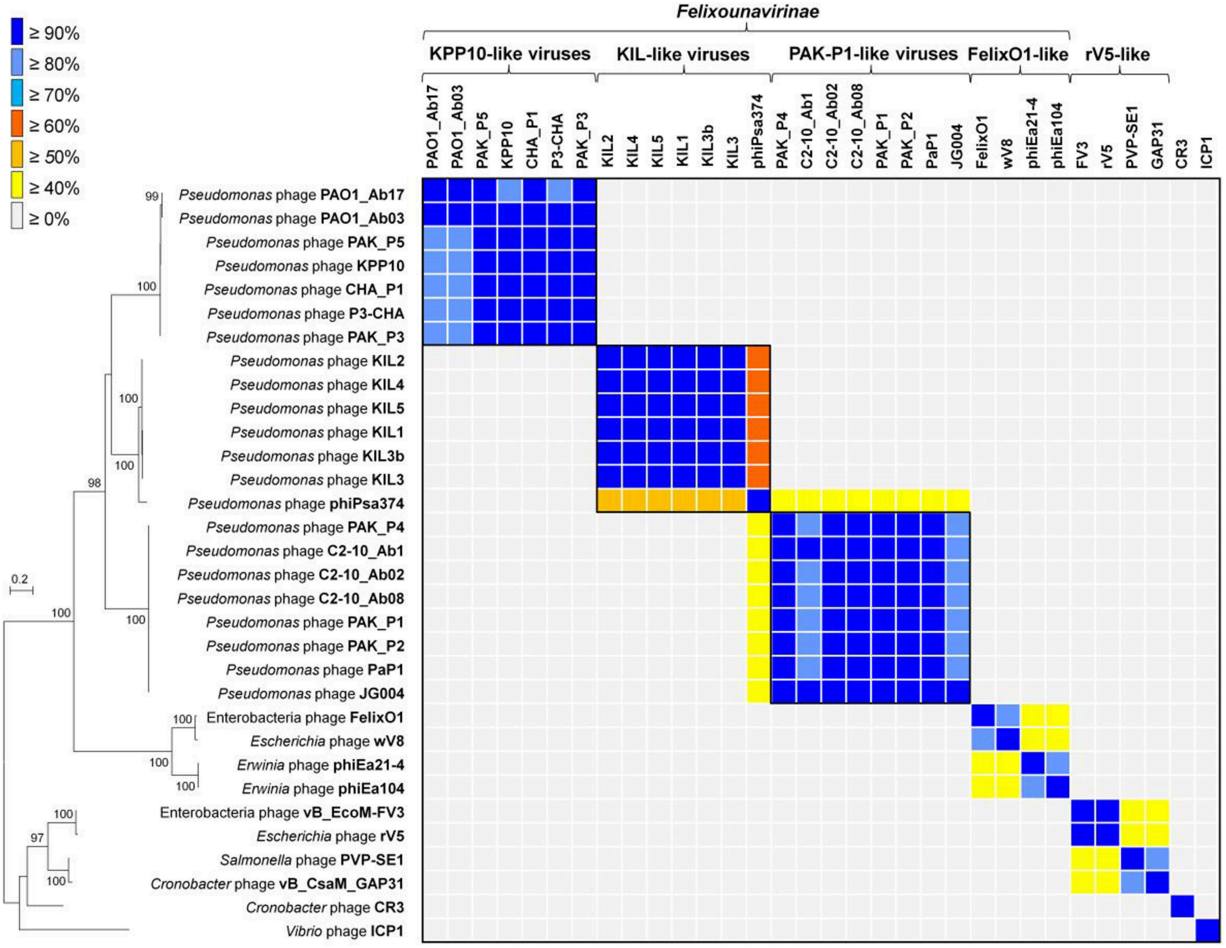
**Phylogenetic and proteome-based relationships of phages KIL1, KIL2, KIL3, KIL4, KIL5, and KIL3b**. Left, maximum-likelihood tree was constructed upon a concatenation of two structural proteins (major capsid protein and baseplate protein) that are common to the 32 phages. The numbers at the branch represent the bootstrap values. Edges with bootstrap values above 75% are represented. The scale bar indicates the number of substitution per site. Right, in the matrix view, the columns and rows correspond to the phages. Each cell indicates the percentage of shared protein families between two phages. Color tags are used to visualize the genomic relatedness based on the proportion of shared protein families.

To further frame these phages in their comparative genomic context, phylogenetic trees were constructed based on the concatenated datasets of two structural genes encoding the major capsid protein and the baseplate protein. These two proteins are shared among the genomes of four clades: the FelixO1-like viruses, PAK_P1-like viruses, and KPP10-like viruses within the *Felixounalikevirus* genus and the rV5-like viruses, as well as phages CR3, ICP1, and our six phage isolates. Each protein was assigned to “Family:virus:314” and “Family:virus:3622” by ACLAME-based clustering (Table S2). Phylogenetic analysis revealed the four clades comprising the KPP10-, PAK_P1-, FelixO1, and rV5-like phages, respectively (Left, Figure 3). Most clades branch deeply and are well supported with bootstrap support values of more than 90%. Within the *Felixounalikevirus* genus, the *Pseudomonas* phages KIL1, KIL2, KIL3, KIL4, KIL5, and KIL3b are grouped into a distinct clade, together with a *Pseudomonas* phage phiPsa374.

To investigate the genome relatedness based on the proportion of conserved homologous proteins between two phages, we performed pairwise comparisons of the 32 phage genomes, which allowed us to assign 5622 protein sequences to 1322 homologous protein families (Table S1). A cut-off value of 40% of homologous proteins was used to assign phage genomes to a putative genus (Lavigne et al., 2008, 2009b). The pattern of proteome-based grouping was highly similar to our phylogenetic results, in which KIL1, KIL2, KIL3, KIL4, KILP5, KIL3b, and phiPsa374 form a conserved group with ≥50% homologous proteins in common to each other's genome (Right, Figure 3). These seven phages are also related to three groups of the *Felixounalikevirus* phages, sharing more than 20% of their homologous proteins, with the PAK_P1-like phages being the closest relatives (average 34% shared proteins). Notably, of these, phiPsa374 shared the highest overlap percentages of the proteomic content (≥40%) with the PAK_P1-like phages.

#### Proteome analysis

Analysis of the proteome of phages KIL3 and KIL5 (representatives of the two clades) by ESI-MS/MS led to the identification of 51 and 67 proteins, respectively (Table S3). For KIL3 and KIL5, respectively 27 proteins and 26 had a predicted function based on homology to other phage proteins. In both phages, most of the detected proteins are early phage proteins or structural phage proteins. The use of non CsCl-purified phages might explain the presence of the non-structural phage proteins since they can remain in the phage lysate after bacterial lysis. Alternately, these proteins may be considered as candidate proteins which may be co-injected during infection. The most abundant proteins based on the number of unique peptides recovered are the tape measure protein, ORF 46 (no predicted function) and the major capsid protein in phage KIL3. Similarly, the most abundant proteins of phage KIL5 are the capsid protein, ORF 46 (no predicted function) and the tail fiber protein (ORF 76). As was seen by Henry et al. (2015) for the related phage PAK_P3, the ORF upstream of the capsid protein had the second highest relative abundance (total number of spectra / Molecular Weight) next to the capsid protein itself, and this in both phages. Although the function of this protein could not be predicted, an association with the capsid protein is suspected. The differences in the number of proteins identified for the two phages could be explained by a difference in protein concentration as the number of peptides recovered for proteins of KIL5 were in general higher than those for KIL3. The identification of these peptides by mass spectrometry confirms the *in silico* ORF predictions. One structural protein, a tail lysozyme (ORF 70), could not be detected in KIL3; however, the corresponding protein in KIL5 (ORF 71) was identified. All other predicted structural proteins could be experimentally confirmed by mass spectrometry.

### Phage therapy

#### Bio-assay on leek leaves

To investigate whether the phages were also capable of lysing their bacterial host in the plant environment, bio-assays were performed on leek leaves.

**Figure 4:**
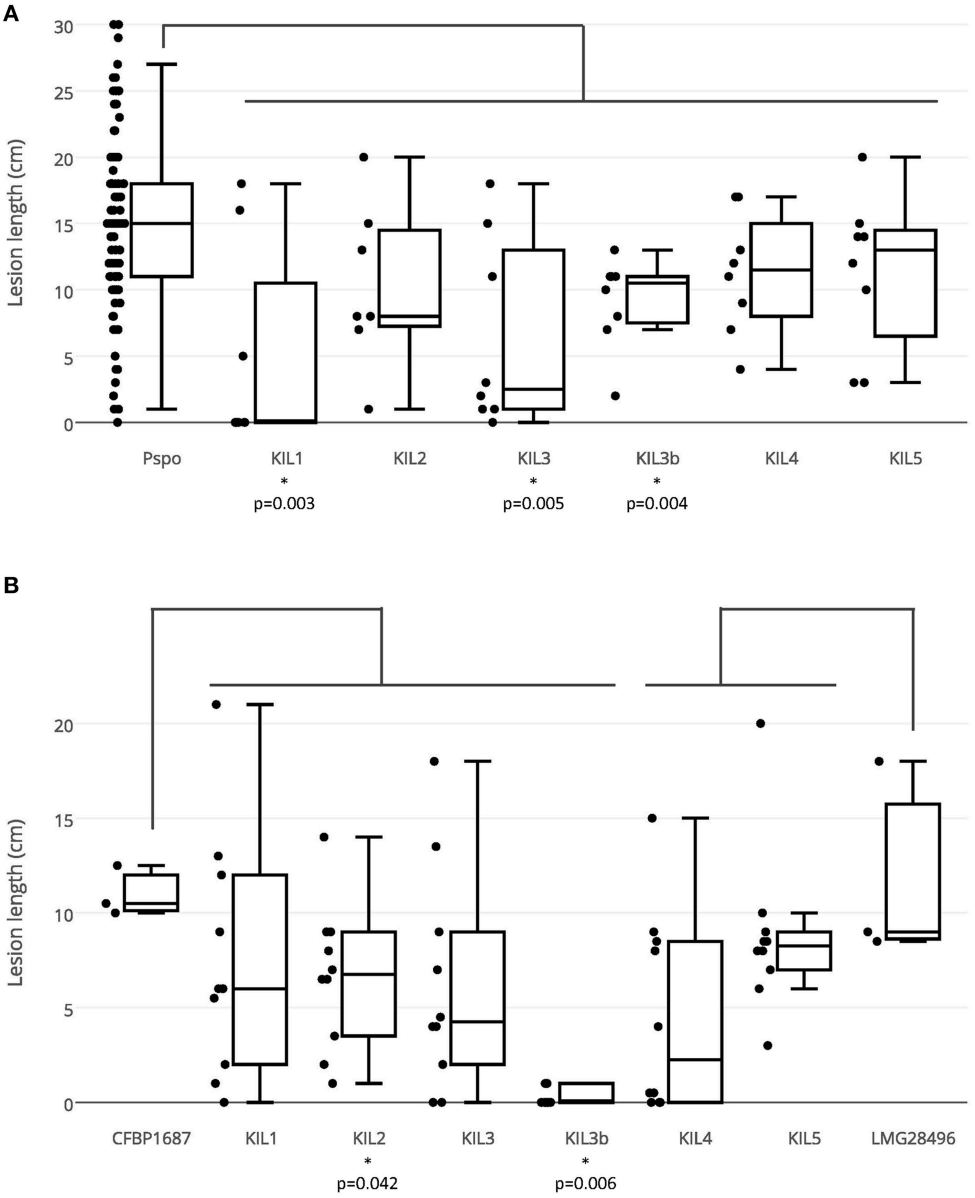
**(A)** Lesion lengths in the first bio-assay in which all 41 *P. syringae* pv. *porri* strains (10^7^ CFU/ml) were inoculated in leek leaves (three inoculated leaves/strain) and all phage types (10^9^ pfu/ml) were co-inoculated with a *P. syringae* pv. *porri* representative (eight inoculations/phage-bacteria combination). **(B)** Lesion lengths in the second bio-assay in which KIL1, KIL2, KIL3, and KIL3b (10^9^ pfu/ml) were co-inoculated with bacterial strain CFBP 1687 (10^6^ CFU/ml) and phages KIL4 and KIL5 with LMG 28496 (10 inoculations/phage-bacteria combination).

In a first bio-assay, the bacterial concentration was chosen to be 10^7^ CFU/ml, the amount of bacteria necessary to produce clear symptoms. For the phages, a relatively high concentration of 10^9^ pfu/ml was used to prove efficacy. All 41 *P. syringae* pv. *porri* strains were inoculated and showed comparable virulence, presented on the left side of the boxplot (Figure 4). Strains CFBP 1687 and CFBP 1770 were chosen as representatives of *P. syringae* pv. *porri* and were used as bacterial host in combination with the different phages. Lesion lengths on the phage treated leaves were compared to the lesion lengths induced by all *P. syringae* pv. *porri* strains used in this study. In general, each phage reduced the mean lesion length compared to the non-phage treated leaves. The reduction in lesion length was significant for phages KIL1, KIL3, and KIL3b with *p*-values of 0.003, 0.005, and 0.004, respectively. Phages KIL2, KIL4, and KIL5 reduced the lesion length, but were not able to prevent bacterial infection completely.

In a second bio-assay, the effect of the phages on a lower bacterial concentration of 10^6^ CFU/ml was tested using ten leaves per phage. CFBP 1687 was used in combination with phages KIL1, KIL2, KIL3, and KIL3b, phages KIL4 and KIL5 were co-inoculated with bacterial strain LMG 28496. Again, lesion lengths of the phage-treated leaves were compared to the lengths produced by their respective host without phage. As expected, lesion lengths were smaller in all treatments compared to the previous assay with higher bacterial concentrations. Only phages KIL2 and KIL3b decreased lesion length significantly compared to their bacterial control, with *p*-values of respectively 0.042 and 0.006. Leaves treated with phage KIL3b showed no symptoms for the most part. The results of this second bio-assay confirm that phages KIL4 and KIL5 are less suitable to reduce lesion length significantly. Only phage KIL3b showed significant reduction in both assays, demonstrating its *in planta* antibacterial effect. The effect of phages KIL1, KIL2, and KIL3 varied between the assays. Nevertheless, it was considered prudent to maintain all phages in a cocktail for subsequent testing in field trials.

#### Field trials

In a first field trial set-up, the potential of phages to protect leek transplants against bacterial infection at planting was tested. Transplants were submerged in a phage solution containing the six phages before planting them in an infested field. This experiment was performed on three different locations in Flanders in the year 2014 (Table 3).

**Table 3:** **Percentage symptomatic plants after 3 months for the field trial at three different locations where 1200 transplants were submerged in phage solution (10^7^ pfu/ml) before planting in a field infested with CFBP 1687**.

**Phage treatment**	**PCG**	**PSKW**	**Inagro**
No	92.5	15.0	23.5
Yes	82.0	18.0	18.0

In the trial at PCG, leek plants of cultivar Kenton (Nunhems) were used. The first symptoms developed 25 days after planting in phage-treated and non-treated plants. Two months later, percentages of diseased plants increased to 82% for the phage-treated plants and 92.5% for the control plants. Later on, symptoms disappeared again in both groups and percentages of diseased plants were again lower 4 months after planting. The same trend was apparent when scoring the percentage of leaf surface affected.

The same experiment was performed in PSKW with cultivar Harston (Nunhems). Two and three months after planting, the disease incidence was measured. Percentages of symptomatic plants varied from 29% for the phage-treated plants and 19% for the control plants after 2 months to 18 and 15% after 3 months, respectively. No significant differences were observed.

In the experiment at Inagro, leek plants of cultivar Harston (Nunhems) were used. No disease incidence was recorded 2 months after planting. After 3 months, measurements reported an infection rate of 23.5% of control plants and 18% for the phage treated plants.

In all three experiments, no significant differences in disease occurrence between treated and untreated plants could be noticed. In the trials performed at PSKW and Inagro, disease incidence remained low indicating that infection was not successful. Conclusions on the efficacy of phage treatment could therefore not be made. From the trial results of PCG we can conclude that phage treatment was not successful in protecting the leek plants from infection when planted in an infested field. However, monitoring at an earlier time-point could be better to detect an effect of the phages. It is also possible that more frequent phage applications are necessary to protect the plants against infection.

In a second field trial, phages were sprayed on artificially infected plants, to test for their potential as a crop protection agent in a later growth stage. First, bacteria were sprayed onto the plants 3 months after planting. The day after, a phage cocktail with the six phages present in a concentration of 10^9^ pfu/ml each was sprayed onto the plants. Results are shown in Table 4.

**Table 4:** **Percentage symptomatic plants after 1 (PCG and PSKW) and 2 months (Inagro) for the field trial with ***P. syringae*** pv. ***porri*** CFBP 1687 (10^**6**^ CFU/ml) and the phage cocktail (10^**9**^ pfu/ml) being sprayed over 1000 leek plants per treatment**.

**Treatment**	**PCG**	**PSKW**	**Inagro**
Untreated	6.0	32.0	19.5
Phage	11.0	38.0	24.0
Bacteria	63.0	42.0	21.5
Bacteria + phage	38.5	30.0	19.0

In the trial at PCG, again cultivar Kenton (Nunhems) was used and phage treatment was repeated four times every 2 weeks. The first disease assessment was performed 19 days after infection and demonstrated a higher infection rate at the bacteria treated plant in relation to the other plants, meaning that infection was successful. One month after infection, infection rate in the bacteria-treated plants increased and a difference trend could be measured for the phage-treated object in comparison with the non-treated infected object with, respectively, 38.5 and 63% symptomatic plants. In subsequent measurements this trend continued although the differences became less apparent. The same trend was demonstrated by the data displaying the affected leaf surface. At all time-points, infection was slightly higher in the phage-treated control compared to the untreated plants. The trial demonstrated that phages could decrease symptom development but were not able to completely stop infection. A higher phage concentration and more frequent applications can possibly lead to a better disease control.

The same trial was performed at PSKW with plants of the cultivar Harston (Nunhems). Phage treatments were applied every week for 8 weeks with half the dose of phages compared to the trial of Kruishoutem, meaning that a concentration of 0.5 × 10^9^ pfu/ml of each phage was used. Disease incidence was measured one, 2 and 3 months after infection but no differences between the plants could be measured. Even the untreated and infected plants showed the same level of symptoms, indicating the possibility that symptoms were masked by natural infection with another pathogen.

The trial performed at Inagro used plants of the cultivar Harston (Nunhems). Five phage applications were carried out: one a week before infection, one the evening of the day during which bacterial infection was performed, and three more weekly applications after infection. Two months after infection disease incidence was measured showing no differences between the four treatments. Even the uninfected and untreated control and the uninfected phage-treated control displayed an infection of respectively 19.5 and 24% indicating the presence of a natural infection that could not be affected by our phages.

Results of the three trials demonstrate that only at PCG conducive conditions for infection with *P. syringae* pv. *porri* were present. From that trial we can conclude that phage application can lead to a reduction in bacterial disease incidence but cannot completely prevent it. Other factors such as natural infections with other pathogens make it difficult to predict the effect and reliability of phage therapy in field trials.

## Discussion

The increase of bacterial blight of leek and the related economical losses attracted our interest in the problem a few years ago leading to an extensive investigation of the causative agent, *P. syringae* pv. *porri*. Previous research divided the Flemish isolates into two groups based on their BOX-PCR fingerprints (Rombouts et al., 2015). Genomic differences among *P. syringae* pv. *porri* strains were also described by Noble et al. (2006), who showed minor differences based on profiles generated with IS50-primer amplification and RFLP of a 16S rDNA fragment. In contrast, these authors as well as Koike et al. (1999) and van Overbeek et al. (2010) did not discriminate different genotypes among their isolates from California, Australia and the Netherland when applying different repetitive-element-PCRs (BOX, REP, and ERIC). This overall bacterial diversity was kept in mind when looking for novel phages capable of lysing *P. syringae* pv. *porri*. Host range analysis of the five phages isolated from soil and the H-mutant phage confirmed the BOX-PCR grouping of bacterial strains, suggesting that differences at the bacterial genome are responsible for the observed host range. Previous genome analysis of a bacterial strain of each group demonstrated that variation was mostly situated in prophage content and mobile genetic elements (Rombouts et al., 2015). No specific element could be found that linked bacterial diversity with the phage host range. In general, genomic differences can affect phage infection at different stages. If those genome differences result in an altered phage receptor, different receptor binding proteins (RBP) capable of recognizing the altered receptor of the five bacterial strains in group one could be present among the phages KIL4 and KIL5. Changes in host range due to a spontaneous mutation in a tail fiber protein (replacement of a positively charged lysine by an uncharged asparagine) were previously reported for two related phages, *Pseudomonas aeruginosa* phages PaP1 and JG004 (Le et al., 2013). However, the exact role of the variation and mutation observed here does not lead to a straightforward hypothesis and requires further investigation. In spite of the overall bacteriophage diversity, all phages we found against *P. syringae* pv. *porri* belong to the *Myoviridae* family. Genome sequence analysis further classified them into a new clade within the *Felixounavirinae* genus.

The phage characteristics analyzed in this study show that all six phages are suitable for use in phage therapy. First, they are strictly virulent, meaning that infection with the phages results in bacterial lysis minimizing the risk of horizontal transfer of pathogenicity genes as is the risk with lysogenic phages (Penadés et al., 2015). Furthermore, by demonstrating a temperature stability from 4 to 37°C and pH stability from pH 4 to 12, they are capable of surviving in the plant environment when applied. However, a disadvantage is their limitation to infect only bacteria grown at temperatures of 28°C or lower. This means that in summer, when temperatures occasionally rise above 28°C bacteria may not be killed by the phages. Probably, the bacterial receptor necessary for phage infection is not expressed at higher temperatures. Temperature dependent infection was previously reported for phage φS1 which is not able to infect *Pseudomonas fluorescens* cells grown at 37°C (Sillankorva et al., 2004). It has been shown that expression of bacterial genes can be temperature dependent. For example, the expression of flagellar components and the surfactant syringafactin is reduced in *P. syringae* strain B728a at a temperature of 30°C, resulting in reduced swimming and swarming motility (Hockett et al., 2013). Host range analysis demonstrates that none of the phages is capable of lysing all tested *P. syringae* pv. *porri* strains, but a combination of different phages in a phage cocktail can cover all strains used. The results indicate that there is some resistance development among *P. syringae* pv. *porri* strain CFBP 1687 when challenged with the bacteriophages KIL3 or KIL4. Although the kill curves indicate that in the first 5 h there is no resistance development, after 72 h an average resistance frequency of around 2 × 10^−6^ was noted. When phages are applied in a phage cocktail, this resistance development is less likely to occur (Gill and Hyman, 2010; Barbosa et al., 2013). The possibility of resistance through lysogenic conversion was excluded, thereby confirming their suitability for phage therapy for this parameter. In addition, development of the host-range mutant phage KIL3b demonstrates that host-range expanding adaptions are possible to counter bacterial resistance, especially because this phage was still able to infect more than half of the resistant bacteria.

To investigate evolutionary relationships of KIL1, KIL2, KIL3, KIL4, and KIL5 with their close relatives, phylogenetic analysis was combined with a whole genome proteomic approach (Lavigne et al., 2008, 2009b). The overall phylogenetic relationships between the concatenated sequences of the major capsid protein and the baseplate proteins of the *Felixounalikevirus* phages are consistent with previous taxonomic description (Henry et al., 2015). The phages KIL1, KIL2, KIL3, KIL4, and KIL5 as well as phiPsa374 can clearly be grouped into a new phylogenetic clade. Their location within the *Felixounalikevirus* genus suggests that the sequences of two structural proteins are highly conserved within their own group and appear to be most closely related to those of the “PAK_P1-like viruses” and “KPP1-like viruses.” In addition, our proteome-based analysis, which is more sensitive and accurate for taxonomic classification for phage genomes (Lavigne et al., 2008), supports the phylogenetic relationships of the *Felixounalikevirus* phages. Given that shared homologs between the phage genomes can be used as an indication of phage members in the same genus (Lavigne et al., 2008, 2009b), we proposed a possible division of the *Felixounalikevirus* genus into the four subclades, which include the “KPP10-like viruses” (phages PAO1_Ab17, PAO1_Ab03, PAK_P5, KPP10, CHA_P1, P3-CHA, and PAK_P3), “PAK_P1-like viruses” (PAK_P4, C2-10_Ab1, C2-10_Ab02, C2-10_Ab-8, PAK_P1, PaP1, and JG004), “FelixO1-like viruses” (FelixO1, wV8, phiEa21-4, and phiEa104), and “KIL-like viruses” (KIL1, KIL2, KIL3, KIL4, KILP5, KIL3b, and phiPsa374). Interestingly, although phage phiPsa374 has been suggested as a member of the PAK_P1-like clade (Frampton et al., 2014; Henry et al., 2015), our results indicate the genome of phiPsa374 aligns with more shared protein families to the KIL-like phages.

No toxin genes, virulence genes or genes related to lysogeny could be discovered in the phage genomes at this time, indicating their suitability for phage therapy. Efficacy of the phages in the plant environment was analyzed with bio-assays in leek leaves. Significant results were only obtained for phages KIL1, KIL2, KIL3, and KIL3b but the broader host range of phages KIL4 and KIL5 led to their incorporation in the phage cocktail for the field trials. When treating transplants with a phage cocktail before planting in an infested field, no significant differences were observed. Spraying of phages on artificially infected plants led to a slightly reduced symptom development in one of the three trials, yet again without statistical significance. Since two trials suffered from natural infections, their impact has most likely masked the effect of the treatments. Although isolation and characterization of phages is reported for several plant pathogens, results of other field trials are scarce. A recent overview has recently been published by Czajkowski (Czajkowski, 2016). A field trial with phages against *Dickeya solani* on potato also reports of phage therapy resulting in minor differences in disease severity (Adriaenssens et al., 2012). In experiments with phages against *X. axonopodis* pvs *citri* and *citrumelo*, significant control of bacterial spot was reported, yet treatment with copper-mancozeb was more effective (Balogh et al., 2008). As other researchers mentioned, open field (and greenhouse) applications with phages encounter many challenges such as UV-light, desiccation, application method, and the need of a large quantities of phages. Especially the phyllosphere is a harsh environment for phages, making leaf diseases difficult to treat (Gill and Abedon, 2003; Goodridge, 2004; Iriarte et al., 2012). This study indicates that phage therapy is able to reduce bacterial blight symptoms in leek, but to improve the efficacy of the phages in the field, phage persistence in the plant phyllosphere should be improved by the use of protective formulations, addition of non-pathogenic phage-propagating bacterial strains or adapting timing and frequency of application as suggested previously (Jones et al., 2007; Balogh et al., 2010).

## Author contributions

Conceived and designed the experiments: SR, AV, JV, MM, RL. Performed the experiments: SR, AV, SV, BD, DV, CA, CV, HJ, YB, JN, JK. Analyzed the data: SR, DV, YB, MM, RL. Contributed reagents/materials/analysis tools: JN, JK, MM, RL. Wrote the paper: SR. All authors read and approved the final manuscript.

### Conflict of interest statement

The authors declare that the research was conducted in the absence of any commercial or financial relationships that could be construed as a potential conflict of interest.
