# Supplementary material for: Characterization of Novel Bacteriophages for Biocontrol of Bacterial Blight in Leek Caused by Pseudomonas syringae pv. porri
Source: Front Microbiol. 2016 Mar 15;7:279. doi: 10.3389/fmicb.2016.00279 (PMC4791379; doi:10.3389/fmicb.2016.00279)
Supplement: Supplementary file 1 [file Table1.DOCX]

**Supplementary Table 1**: Homopolymeric stretches in the phage genomes.

|  | GGCGGC –G-stretch- TCAATT | TTGATT –G-stretch- TGTTAT | TGAGCT – A-stretch- GTGTTG | GGTAGA – C-stretch- ATAGGC |
| --- | --- | --- | --- | --- |
| KIL1 | 6 G’s | 13 G’s | 14 A’s | / |
| KIL2 | 6 G’s | / | 14 A’s | / |
| KIL3 | 6 G’s | 13 G’s | 14 A’s | / |
| KIL4 | 13 G’s | 11 G’s | 24 A’s | 13 C’s |
| KIL5 | 13 G’s | 11 G’s | 24 A’s | 13 C’s |
| KIL3b | 13 G’s | 13 G’s | 33 A’s | / |
