# Supplementary material for: Characterization of Novel Bacteriophages for Biocontrol of Bacterial Blight in Leek Caused by Pseudomonas syringae pv. porri
Source: Front Microbiol. 2016 Mar 15;7:279. doi: 10.3389/fmicb.2016.00279 (PMC4791379; doi:10.3389/fmicb.2016.00279)
Supplement: Supplementary file 3 [file Table3.DOCX]

**Supplementary Table 3**: Characteristics of the ESI-MS/MS identified proteins of phages KIL3 and KIL5

| ORF | **Putative protein function** | **Protein size (kDa)** | **N° of unique peptides recovered** | **Protein coverage^a^** | **ORF** | **Putative protein function** | **Protein size (kDa)** | **N° of unique peptides recovered** | **Protein coverage^a^** |
| --- | --- | --- | --- | --- | --- | --- | --- | --- | --- |
| 3 | Hypothetical protein | 16.21 | 1 | 8.82% | **3** | Hypothetical protein | 13.35 | 2 | 18.96% |
| 18 | Putative DNA-binding protein | 25.82 | 3 | 17.04% | **20** | Hypothetical protein | 25.82 | 5 | 25.56% |
| 21 | Putative DNA ligase | 46.25 | 12 | 37.10% | **21** | Hypothetical protein | 25.51 | 5 | 28.03% |
| 30 | Putative phage antirepressor protein | 29.64 | 6 | 29.69% | **23** | Putative DNA ligase | 46.18 | 20 | 65.98% |
| 31 | Putative serine protease | 23.21 | 6 | 48.33% | **29** | Hypothetical protein | 14.79 | 1 | 9.67% |
| 32 | Putative phosphate starvation inducible protein, PhoH | 28.29 | 9 | 53.27% | **30** | Putative phage antirepressor protein | 29.64 | 12 | 62.71% |
| 37 | Hypothetical protein | 12.17 | 1 | 11.75% | **31** | Hypothetical protein | 23.21 | 7 | 51.17% |
| 42 | Hypothetical protein | 30.58 | 2 | 10.07% | **32** | Putative phosphate starvation inducible protein, PhoH | 28.29 | 10 | 56.38% |
| 44 | Putative terminase large subunit | 51.97 | 9 | 23.49% | **33** | Hypothetical protein | 13.77 | 1 | 13.58% |
| 45 | Hypothetical protein | 21.91 | 1 | 6.02% | **34** | Hypothetical protein | 21.25 | 4 | 38.31% |
| 46 | Hypothetical protein | 54.78 | 24 | 68.88% | **38** | Hypothetical protein | 12.17 | 4 | 49.72% |
| 47 | Putative methyltransferase | 17.10 | 1 | 12.22% | **42** | Hypothetical protein | 30.62 | 4 | 26.58% |
| 48 | Hypothetical protein | 35.57 | 9 | 38.66% | **44** | Putative terminase large subunit | 52.22 | 11 | 31.60% |
| 49 | Hypothetical protein | 14.57 | 5 | 67.20% | **46** | Hypothetical protein | 54.76 | 26 | 72.72% |
| 50 | Putative major capsid protein | 39.84 | 16 | 62.40% | **47** | Putative phage protease XkdF | 18.45 | 6 | 48.30% |
| 51 | Hypothetical protein | 22.60 | 3 | 23.85% | **48** | Hypothetical protein | 35.59 | 12 | 56.86% |
| 53 | Hypothetical protein | 13.97 | 5 | 53.55% | **49** | Putative structural protein | 14.57 | 6 | 78.53% |
| 54 | Hypothetical protein | 28.64 | 4 | 16.13% | **50** | Putative capsid protein | 39.87 | 23 | 85.81% |
| 55 | Hypothetical protein | 23.06 | 6 | 42.93% | **51** | Hypothetical protein | 22.60 | 4 | 27.74% |
| 57 | Putative structural protein | 45.76 | 14 | 51.20% | **53** | Hypothetical protein | 13.97 | 3 | 37.02% |
| 59 | Hypothetical protein | 19.92 | 2 | 13.25% | **54** | Hypothetical protein | 22.26 | 5 | 37.56% |
| 61 | Hypothetical protein | 19.14 | 1 | 8.62% | **56** | Putative structural protein | 45.76 | 14 | 52.88% |
| 64 | Hypothetical protein | 11.10 | 2 | 20.81% | **60** | Putative structural protein | 18.81 | 3 | 35.68% |
| 65 | Putative tape measure protein | 87.69 | 24 | 38.01% | **61** | Hypothetical protein | 22.93 | 1 | 11.03% |
| 66 | Hypothetical protein | 29.54 | 5 | 21.60% | **62** | Hypothetical protein | 19.15 | 2 | 13.78% |
| 57 | Hypothetical protein | 13.62 | 1 | 10.50% | **64** | Hypothetical protein | 13.01 | 2 | 33.83% |
| 68 | Putative tail protein | 35.11 | 7 | 33.84% | **65** | Hypothetical protein | 11.09 | 3 | 47.62% |
| 69 | Putative baseplate protein | 27.97 | 5 | 26.35% | **66** | Putative tape measure protein | 85.30 | 16 | 25.28% |
| 71 | Putative baseplate protein | 53.46 | 11 | 41.36% | **67** | Hypothetical protein | 29.86 | 6 | 26.15% |
| 72 | Hypothetical protein | 26.79 | 4 | 27.51% | **68** | Hypothetical protein | 13.62 | 1 | 10.50% |
| 73 | Tail fiber protein | 72.00 | 14 | 40.49% | **69** | Hypothetical protein | 35.11 | 9 | 41.05% |
| 75 | Tail fiber protein | 51.18 | 13 | 46.21% | **70** | Putative baseplate protein | 27.98 | 4 | 21.23% |
| 76 | Endolysin | 20.84 | 2 | 19.53% | **71** | Putative tail lysozyme | 16.41 | 6 | 51.61% |
| 83 | Putative RNA ligase | 43.55 | 4 | 12.38% | **72** | Putative baseplate protein | 53.43 | 10 | 38.50% |
| 91 | Hypothetical protein | 24.42 | 6 | 38.74% | **73** | Hypothetical protein | 26.79 | 8 | 47.62% |
| 97 | Putative DNA primase | 106.89 | 16 | 20.89% | **74** | Putative tail fiber protein | 72.03 | 11 | 31.00% |
| 99 | Putative DNA polymerase | 124.51 | 15 | 17.40% | **75** | Hypothetical protein | 16.04 | 2 | 17.83% |
| 102 | Hypothetical protein | 15.96 | 3 | 34.47% | **76** | Tail fiber protein | 51.17 | 20 | 82.12% |
| 103 | Hypothetical protein | 35.78 | 4 | 11.99% | **77** | Endolysin | 20.84 | 7 | 50.68% |
| 108 | Putative restriction endonuclease | 24.92 | 5 | 33.11% | **79** | Hypothetical protein | 15.53 | 2 | 20.54% |
| 109 | Putative exodeoxyribonuclease | 39.26 | 3 | 9.81% | **80** | Hypothetical protein | 17.65 | 1 | 8.72% |
| 110 | Hypothetical protein | 23.02 | 4 | 21.50% | **82** | Hypothetical protein | 13.02 | 3 | 34.64% |
| 111 | Putative exonuclease | 36.24 | 4 | 17.60% | **84** | Putative RNA ligase | 43.55 | 11 | 41.42% |
| 112 | Hypothetical protein | 13.92 | 1 | 11.06% | **90** | Hypothetical protein | 14.14 | 2 | 23.34% |
| 116 | Hypothetical protein | 22.07 | 2 | 11.96% | **92** | Hypothetical protein | 24.45 | 12 | 87.29% |
| 118 | Putative HNH endonuclease | 18.12 | 2 | 12.14% | **98** | Putative DNA primase/helicase | 106.77 | 19 | 27.61% |
| 119 | Hypothetical protein | 13.50 | 1 | 8.96% | **99** | Putative DNA polymerase | 126.41 | 14 | 16.10% |
| 120 | Putative ribonucleotide-diphosphate beta subunit | 16.65 | 1 | 10.57% | **102** | Hypothetical protein | 15.95 | 2 | 28.97% |
| 121 | Putative ribonucleotide-diphosphate alfa chain | 65.20 | 10 | 23.28% | **103** | Hypothetical protein | 35.66 | 12 | 51.20% |
| 122 | Putative DNA adenine methyltransferase | 30.55 | 3 | 14.04% | **108** | Putative exodeoxyribonuclease | 41.82 | 4 | 11.05% |
| 130 | Putative pyrophosphatase | 15.72 | 1 | 9.80% | **110** | Putative HNH endonuclease | 22.80 | 4 | 20.26% |
|  |  |  |  |  | **113** | Putative metallo-dependent phosphatase | 28.73 | 6 | 36.38% |
|  |  |  |  |  | **115** | Hypothetical protein | 13.92 | 2 | 30.03% |
|  |  |  |  |  | **119** | Hypothetical protein | 22.05 | 3 | 18.95% |
|  |  |  |  |  | **120** | Putative thymidylate synthase | 39.22 | 2 | 8.69% |
|  |  |  |  |  | **121** | Hypothetical protein | 18.12 | 3 | 16.39% |
|  |  |  |  |  | **122** | Hypothetical protein | 13.26 | 1 | 14.94% |
|  |  |  |  |  | **123** | Putative ribonucleotide-diphosphate beta subunit | 25.08 | 1 | 4.83% |
|  |  |  |  |  | **124** | Putative ribonucleotide-diphosphate alpha subunit | 65.20 | 8 | 16.70% |
|  |  |  |  |  | **125** | Putative DNA adenine methyltransferase | 30.55 | 4 | 19.09% |
|  |  |  |  |  | **133** | Hypothetical protein | 15.69 | 4 | 41.36% |
|  |  |  |  |  | **136** | Hypothetical protein | 16.26 | 2 | 29.09% |
|  |  |  |  |  | **137** | Hypothetical protein | 32.55 | 3 | 10.81% |
|  |  |  |  |  | **139** | Hypothetical protein | 38.46 | 4 | 13.44% |
|  |  |  |  |  | **141** | Hypothetical protein | 18.74 | 1 | 12.33% |
|  |  |  |  |  | **145** | Hypothetical protein | 9.04 | 1 | 18.26% |
|  |  |  |  |  | **150** | Hypothetical protein | 49.53 | 3 | 9.77% |

^a^ Coverage of the protein sequence by unique peptides recovered during ESI-MS/MS
