# Supplementary material for: Characterization of Novel Bacteriophages for Biocontrol of Bacterial Blight in Leek Caused by Pseudomonas syringae pv. porri
Source: Front Microbiol. 2016 Mar 15;7:279. doi: 10.3389/fmicb.2016.00279 (PMC4791379; doi:10.3389/fmicb.2016.00279)
Supplement: Supplementary file 4 [file DataSheet1.DOCX]

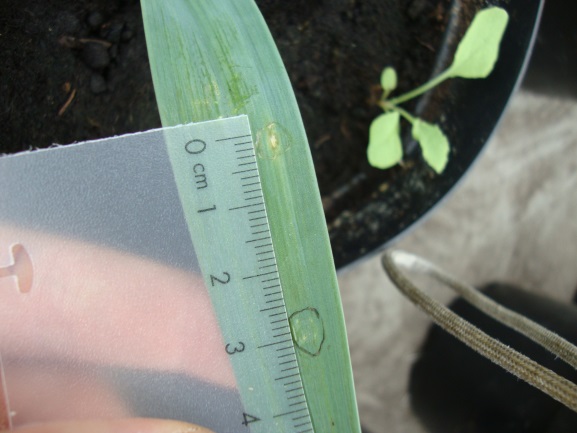


Supplementary Figure 1: Set-up of the leek bio-assay. First, 0.1 ml bacterial suspension with a concentration of 10^7^ CFU/ml was injected with a syringe into the leave. Subsequently, 3 cm above the first injection spot, 0.1 ml of the phage suspension with a concentration of 10^9^ pfu/ml was injected.
