# Supplementary material for: Characterization of Novel Bacteriophages for Biocontrol of Bacterial Blight in Leek Caused by Pseudomonas syringae pv. porri
Source: Front Microbiol. 2016 Mar 15;7:279. doi: 10.3389/fmicb.2016.00279 (PMC4791379; doi:10.3389/fmicb.2016.00279)
Supplement: Supplementary file 5 [file DataSheet2.DOCX]

Supplementary Figure 2: Killing curves of phages KIL3 and KIL5, representing the two phage clades, demonstrating the decrease of OD_600nm_ over time at different MOI’s.
